# The Conserved Macrodomain Is a Potential Therapeutic Target for Coronaviruses and Alphaviruses

**DOI:** 10.3390/pathogens11010094

**Published:** 2022-01-14

**Authors:** Anthony K. L. Leung, Diane E. Griffin, Jürgen Bosch, Anthony R. Fehr

**Affiliations:** 1 Department of Biochemistry and Molecular Biology, Bloomberg School of Public Health, Johns Hopkins University, Baltimore, MD 21205, USA; 2 Department of Oncology, School of Medicine, Johns Hopkins University, Baltimore, MD 21205, USA; 3 McKusick-Nathans Department of Genetic Medicine, School of Medicine, Johns Hopkins University, Baltimore, MD 21205, USA; 4 Department of Molecular Biology and Genetics, School of Medicine, Johns Hopkins University, Baltimore, MD 21205, USA; 5 W. Harry Feinstone Department of Molecular Microbiology and Immunology, Bloomberg School of Public Health, Johns Hopkins University, Baltimore, MD 21205, USA; 6 Center for Global Health and Diseases, Case Western Reserve University, Cleveland, OH 44106, USA; jxb745@case.edu; 7 InterRayBio, LLC, Cleveland, OH 44106, USA; 8 Department of Molecular Biosciences, University of Kansas, Lawrence, KS 66045, USA

**Keywords:** coronavirus, alphavirus, SARS-CoV-2, macrodomain, ADP-ribosylation, ADP-ribosylhydrolase, therapeutics

## Abstract

Emerging and re-emerging viral diseases pose continuous public health threats, and effective control requires a combination of non-pharmacologic interventions, treatment with antivirals, and prevention with vaccines. The COVID-19 pandemic has demonstrated that the world was least prepared to provide effective treatments. This lack of preparedness has been due, in large part, to a lack of investment in developing a diverse portfolio of antiviral agents, particularly those ready to combat viruses of pandemic potential. Here, we focus on a drug target called macrodomain that is critical for the replication and pathogenesis of alphaviruses and coronaviruses. Some mutations in alphavirus and coronaviral macrodomains are not tolerated for virus replication. In addition, the coronavirus macrodomain suppresses host interferon responses. Therefore, macrodomain inhibitors have the potential to block virus replication and restore the host’s protective interferon response. Viral macrodomains offer an attractive antiviral target for developing direct acting antivirals because they are highly conserved and have a structurally well-defined (druggable) binding pocket. Given that this target is distinct from the existing RNA polymerase and protease targets, a macrodomain inhibitor may complement current approaches, pre-empt the threat of resistance and offer opportunities to develop combination therapies for combating COVID-19 and future viral threats.

## 1. Introduction

Emerging and re-emerging viral diseases pose continuous public health threats that have increased with globalization, population growth, urbanization, and climate change. These disease-causing viruses range from zoonotic and sexually transmitted viruses such as human immunodeficiency virus (HIV), to vector-borne viruses such as Zika virus and chikungunya virus (CHIKV), to respiratory viruses such as H1N1 influenza virus and severe acute respiratory syndrome coronavirus 2 (SARS-CoV-2). The rapid mutation of these RNA viruses can facilitate infection of new hosts, improve transmission, and increase virulence with sudden spread into new regions. The medical, social, and political consequences of these emerging infections are global but often disproportionately affect resource-poor countries, resulting in unequal social, health system, and economic burdens on these populations.

Effective control requires a combination of non-pharmacologic interventions, treatment with antiviral drugs and antibodies, and prevention with vaccines. Experience with the COVID-19 pandemic and other recently emergent viral infections has exposed a lack of effective treatments capable of protecting humans from the devastating effects of highly deadly and contagious viral pathogens. This gap in preparedness has been due, in large part, to a lack of investment in development of antiviral agents for classes of viruses that were only “potential threats” and, if they did emerge, often caused only acute disease and were quickly contained. When SARS-CoV-2 emerged, this dearth of therapeutics was tragically exposed. Therefore, a multi-pronged investment in development of multiple classes of direct-acting antiviral drugs effective against emergent RNA viruses is crucial to prepare for the next epidemic/pandemic. 

Coronaviruses, once thought to be mere contributors to the common cold, have now caused three notable epidemics of life-threatening disease in the last two decades: SARS-CoV in 2002–2003, Middle East respiratory syndrome coronavirus (MERS-CoV) in 2012–2015, and the current pandemic of SARS-CoV-2. All of these viruses are capable of causing respiratory infection that can result in severe disease and even death in a high proportion of those infected [1]. Currently, SARS-CoV-2 is the major public health challenge worldwide, with more than 5 million deaths, accompanied by prolonged destabilizing consequences for the global economy, life-expectancy, and education. The COVID-19-induced global recession resulted in an economic contraction of 3.5% in 2020, with the most pronounced downturn in the poorest countries. Furthermore, disruptions to health care systems have increased deaths from other causes and hindered delivery of routine health care services, such as immunizations. Another class of viruses with pandemic potential are the mosquito-borne alphaviruses. Although previously geographically restricted, these viruses continue to expand into new regions to cause epidemics of rash, arthritis, and encephalitis [2,3]. The past 50 years have seen a dramatic emergence/re-emergence of epidemic arboviral diseases, with 3.6 billion people (nearly half the world population) living in urban areas with exposure to efficient *Aedes aegypti* mosquito vectors [4]. In addition to incapacitating acute disease, a high proportion of individuals infected with arthritis-causing alphaviruses, such as CHIKV, Sindbis virus (SINV), and Mayaro virus (MAYV), develop chronic joint pain [5,6]. Although infectious virus is cleared promptly after most acute RNA virus infections, including those caused by alphaviruses and coronaviruses, viral RNA often persists, accompanied by ongoing immune stimulation that may contribute to prolonged symptoms [5,7,8,9,10,11,12,13]. Therefore, lack of effective treatment results not only in deaths due to acute disease but also in prolonged disability that could likely be prevented with antiviral treatment [14,15]. Availability of effective antiviral drugs for both coronavirus and alphavirus infections would decrease hospitalizations, deaths, and long-term disability, with benefits for individuals and society. A common feature of these virus families that offers a potential target for antiviral drug development is the highly conserved macrodomain.

## 2. Macrodomains Represent a Unique Target for Pathogens of Pandemic Potential

Few antivirals are available for treatment of SARS-CoV-2 [16], and no treatments are available for infection with any alphavirus. Antiviral drug development for coronaviruses has mostly focused on nucleoside analogs as inhibitors of RNA polymerase function (e.g., remdesivir or molnupiravir) and peptide analogs that inhibit viral proteases (e.g., nirmatrelvir). Likewise, preliminary evaluation of drugs that inhibit alphavirus replication have most commonly targeted the proteases or polymerase [17,18]. However, there remains a substantial need for developing additional antivirals with novel targets. Identifying distinct targets that require different antiviral mechanisms may complement current approaches, pre-empt the threat of resistance, and offer opportunities to develop combination therapy. Both viral families contain a highly conserved macrodomain that is critical for viral replication and virulence, making it an attractive therapeutic target [19,20]. Macrodomain inhibitors, if successfully developed, could be significant weapons in the future antiviral armamentarium to combat these pathogenic viruses with epidemic potential. Here, we summarize the role of the macrodomain in viral replication and virulence and review the current efforts to develop macrodomain inhibitors as direct-acting antivirals. 

## 3. The Biochemistry of Macrodomains

**Figure 1:**
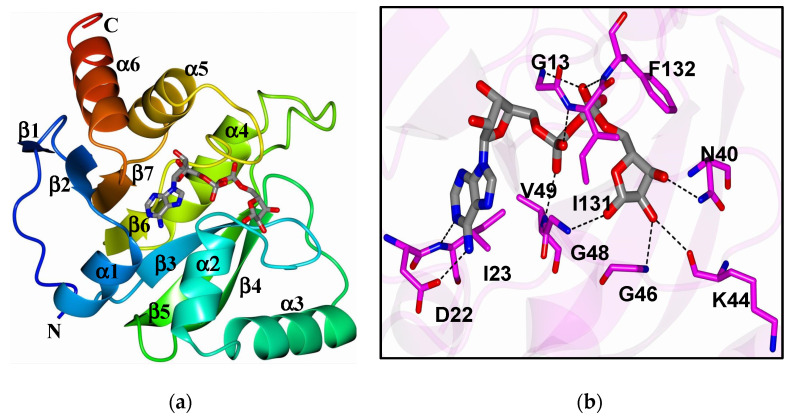
(**a**) The structure of SARS-CoV-2 macrodomain complexed with ADP-ribose (6WOJ); (**b**) hydrogen bonds (dashed lines) between amino acids in the binding pocket and ADP-ribose. Obtained from Alhammad et al., 2020.

ADP-ribosylation is a post-translational modification catalyzed by ADP-ribosyltransferases (ARTs, also known as PARPs [21]) that transfer an ADP-ribose moiety from NAD^+^ onto target proteins [22]. The ADP-ribose molecule is transferred as a single unit of mono-ADP-ribose (MAR) or as consecutively attached single units of MAR through glycosidic bonds to preceding ADP-ribose units covalently to form a poly-ADP-ribose (PAR) chain. A macrodomain is a conserved protein fold, existing either as a single protein or embedded within a larger protein, capable of binding to and, in some cases, reversing this modification [19,20,23]. The macrodomain structure consists of a three-layered α/β/α fold and a conserved ADP-ribose binding pocket (Figure 1a,b) [24]. 

These macrodomains are identified in all kingdoms of life [22], including a subset of plus-strand RNA viruses: alphaviruses, coronaviruses, rubella virus, and hepatitis E virus (HEV) [19,20,23]. Viral macrodomains bind to ADP-ribose, its derivatives, and protein-conjugated ADP-ribose. Viral macrodomains are highly conserved in the nonstructural proteins of both alphaviruses and coronaviruses and belong to the MacroD subclass that has ADP-ribosylhydrolase activity [19,25,26,27,28,29,30]. All alphaviruses contain a single macrodomain in the N-terminal portion of nonstructural protein 3 (nsP3) while the highly pathogenic β-coronaviruses SARS-CoV, SARS-CoV-2, and MERS-CoV contain two to three tandem macrodomains in their nsP3, but only the first (Mac1) possesses ADP-ribosylhydrolase activity [31]. The coronavirus and alphavirus macrodomains are primarily MAR-hydrolases, although alphavirus and HEV macrodomains may also have PAR-hydrolase activity, especially when coupled with other proteins [27,32,33]. Therefore, these viral macrodomains, while conserved across different viral families, may have distinct functions. 

## 4. Viral Macrodomains Are Critical for Viral Replication and Disease Pathogenesis 

Some mutations in the ADP-ribose binding regions of coronavirus, alphavirus, and HEV macrodomains are not tolerated and viruses cannot replicate, indicating an essential function for this domain [28,34,35,36]. Alphavirus macrodomain mutants without binding or hydrolase activity are not viable due to defects in initiation of infection and viral RNA synthesis [34,35]. In addition, hydrolase activity is critical later in infection for translation of the sub-genomic RNA to produce the viral structural proteins and for disruption of stress granules, which are enriched with translation initiation factors [28,33,34]. 

**Figure 2:**
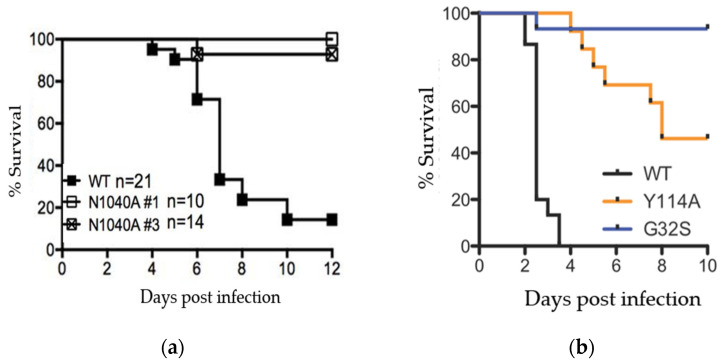
SARS-CoV and CHIKV macrodomain activity is required for viral pathogenesis. (**a**) Female Balb/C mice were infected with a lethal dose of SARS-CoV and equivalent amount of 2 separate clones of macrodomain mutant (N1040A) virus and monitored for survival over 12 days. Data from Fehr et al., 2016; (**b**) 2-day old CD-1 mice (N = 24–28/group) were infected with CHIKV or nsP3 macrodomain mutants Y114A and G32S and monitored for survival over 10 days. Data from McPherson et al., 2017.

Other viruses without macrodomain activity, such as SARS-CoV N1040A, can replicate normally in some tissue culture cells, but these mutants are attenuated in mice [37]. Studies in animals have shown that macrodomain ADP-ribosylhydrolase activity is critical for both coronavirus and alphavirus pathogenesis (e.g., Figure 2a,b) [20,38]. These data indicate that, while there may be virus- and cell-type specific differences in macrodomain function, the macrodomain is universally necessary for virus virulence. 

The coronavirus macrodomain is also required for full repression of the interferon (IFN) response during infection. Mouse hepatitis virus (MHV) and SARS-CoV macrodomain mutant viruses increase innate immune responses following infection [37,39]. Furthermore, it was demonstrated in a co-infection model that this antiviral response helped protect mice from a lethal SARS-CoV infection [37]. Importantly, an early IFN response after infection is critical for its protective effects. Early administration of IFN-I or IFN-III is protective in mouse models of SARS-CoV and MERS-CoV infection, but administration of IFN in the later stages of infection is not [40,41]. Unlike the wild-type virus which suppresses IFN response, macrodomain mutant virus infection elicits IFN induction in the very early stages of infection [37,39]. Thus, macrodomain inhibitors have the potential to restore the host’s protective early and robust IFN response in addition to blocking virus replication. 

## 5. Viral Macrodomain Counteracts Host Antiviral Responses Mediated through PARPs 

Several PARPs are induced by virus infection and by IFN, indicating a role for PARPs and ADP-ribosylation in the antiviral response [39]. Amongst the 17 PARPs in humans, 4 are capable of adding PAR (PARPs 1, 2, 5a, and 5b), 11 add MAR (PARPs 3, 4, 6, 7, 8, 10, 11, 12, 14, 15, and 16), and 2 are catalytically inactive by themselves (PARPs 9 and 13) [21]. PARPs 9, 12, 13, and 14 are amongst the 62 IFN-stimulated genes conserved across vertebrates as part of the innate response to infection [42]. Induction of PARPs is observed in the brains of alphavirus and MHV-infected mice and simian immunodeficiency virus (SIV)-infected macaques, in SARS-CoV-2-infected human lung and bronchial cells, ferrets, and COVID-19 autopsy samples [35,43,44], and likely many other viral infection models. 

Several pieces of evidence indicate that some PARPs may be critical for the host antiviral response [20,45]. Overexpression of PARPs 7, 10, and 12 strongly represses the replication of several classes of RNA viruses [46,47]. More recently, PARP11 and PARP12 were found to cooperatively inhibit Zika virus replication by ADP-ribosylating non-structural proteins, which targeted them for degradation [48,49]. Furthermore, PARP12 was identified in a screen for proteins that interact with SARS-CoV-2 genomic RNA, and PARP12 knockdown enhanced replication of SARS-CoV-2 in Calu-3 cells [50]. In addition, PARP7 and PARP11 ADP-ribosyltransferase activity reduces IFN signaling, resulting in enhanced replication of influenza virus and HSV-1 [51,52]. 

**Figure 3:**
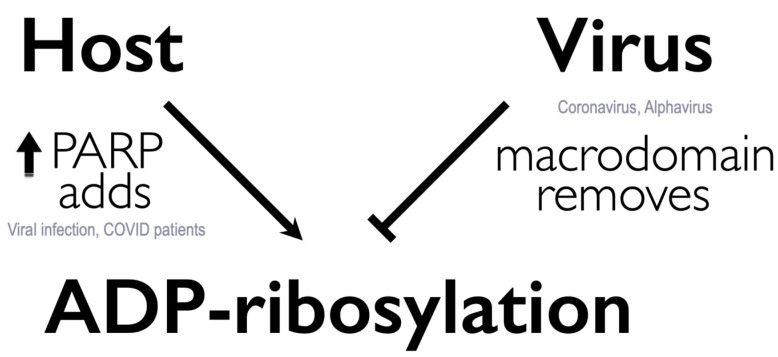
Working model: arms-race on ADP-ribosylation between host PARPs and virus macrodomains.

Most virus-induced PARPs add MAR to proteins, and viral macrodomains primarily bind and remove MAR from protein. Therefore, one intriguing hypothesis is that viruses may circumvent host defenses or regulate replication by binding or removing specific classes of ADP-ribosylation (Figure 3). Indeed, inhibition of PARP activity with 3-AB enhances the replication of a MHV macrodomain mutant virus (N1347A), indicating that the macrodomain counters PARP activity [39]. Similarly, knockdown of PARP12 or PARP14 partially restored the mutant virus replication, demonstrating the ability of these MAR-adding PARPs to restrict MHV replication [39]. On the contrary, enhancing PARP activity by supplementing cells with NAD^+^ precursors, such as nicotinamide riboside, further restricts the replication of these mutant viruses [44], supporting the critical importance of ADP-ribosylation removal by coronaviruses.

## 6. High Conservation of the Viral Macrodomain Indicates the Potential to Develop Broad Spectrum Antivirals 

**Figure 4:**
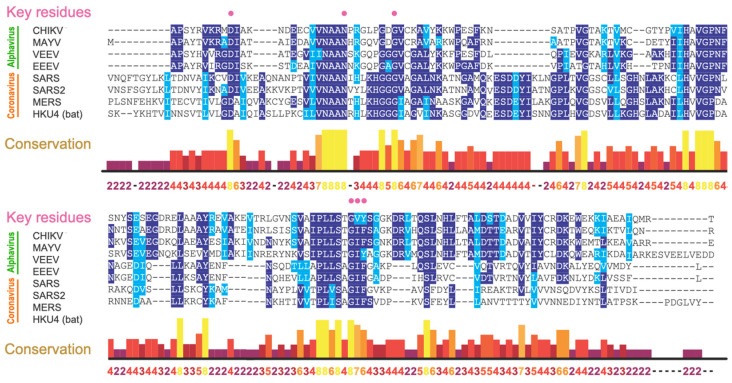
Amino acid analyses revealed high conservation of key residues responsible for macrodomain ADP-ribosylhydrolase activities (pink circles). Alphavirus: CHIKV; MAYV, VEEV, EEEV; Coronavirus: SARS-CoV (SARS), SARS-CoV-2 (SARS2), MERS-CoV (MERS), and a bat coronavirus (HKU4).

The macrodomain is highly conserved in all coronaviruses, including all seven human coronaviruses, with 100% conservation of key residues critical for ADP-ribosylhydrolase activity (Figure 4). These key residues are also conserved in both New World encephalitic and Old World arthritic alphaviruses, such as CHIKV, MAYV, and eastern, western, and Venezuelan equine encephalitis viruses (EEEV, WEEV, and VEEV). The conservation extends to animal reservoir coronaviruses that pose future threats of zoonotic disease. 

Mutagenesis has revealed that structural and amino acid configurations of the macrodomain ADP-ribose binding site are critical for viral replication and virulence. Some mutant viruses are completely non-viable. For example, no mutant viruses can be recovered from cells transfected with CHIKV RNA with mutations of D10A, G32E, G112E, or R144A, and recovered viruses are all reverted to wild type [28]. Similarly, SINV with an N10A mutation is non-viable, and recovered viruses are reverted to the wild type, mutated to D or T, or have developed an A31G compensatory mutation [53]. A similar phenomenon is observed in coronaviruses: most recovered G1439V mutant MHVs are reverted to wild type or have developed a compensatory mutation, A1438T, and the D1329A/N1347A double mutant cannot be recovered [36]. Because these residues line ADP-ribose binding sites, these data indicate strong selective pressure to maintain the macrodomain structural and amino acid configurations. Consistent with this premise, our recent genomic analyses of 440,212 SARS-CoV-2 sequences, including those from all variants of concern, revealed that key residues for binding ADP-ribose remain constant [54]. The high conservation observed suggests the potential of developing antivirals targeting particular, or even across, virus families.

## 7. Structural and Biochemical Data Indicate Feasibility of Developing Macrodomain-Targeted Antivirals 

**Figure 5:**
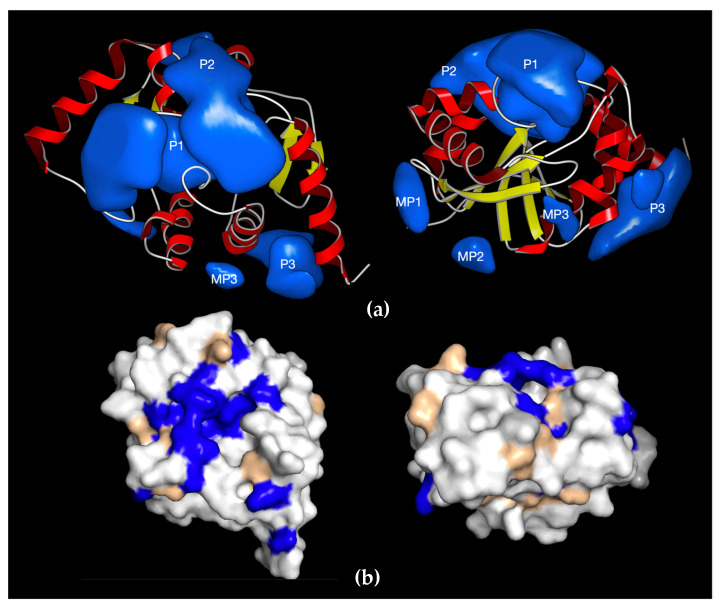
Druggable pockets of SARS-CoV-2 macrodomain and similarities with human MacroD2. (**a**) Ribbon representation of SARS-CoV-2 macrodomain with surface representation of the druggable pockets (P1, P2, P3). Additional minor pockets MP1, MP2, and MP3 are generally too small to be considered a good exploitable binding site as their volume is <200 Å^3^ and would only allow small fragments to bind with typically low inhibitory potential. The right panel shows a 90°-rotated view along two axes; (**b**) identities between SARS-CoV-2 Mac1 macrodomain and the closest human homolog MacroD2 are shown in blue, conserved residues in wheat, and different residues in white. Views in b are identical to the orientation in a.

One advantage of choosing the macrodomain as a drug target is that over 500 structures have been deposited in the Protein Data Bank, including 314 from viruses and 130 from humans. Cross-comparison of this rich structural dataset provides us with foundational information on the commonalities and differences across virus and human macrodomains. Structural analyses of SARS-CoV-2 and CHIKV macrodomains revealed three defined druggable “pockets” near the active site as potential targets for small molecule inhibitors (Figure 5a) [32,55,56,57]. The largest pocket P1 is where ADP-ribose binds, whereas adjacent pockets P2 and P3 could be explored for structure-based drug discovery. Several groups, using computational docking, fragment-based screens, thermal shift assays, and crystallographic screening, independently identified several fragments/compounds, including the metabolite of remdesivir GS-441524, that bind to the SARS-CoV-2 macrodomain [58,59,60,61,62,63,64,65,66,67]. Furthermore, using a displacement assay, some fragments identified in crystallographic screening inhibited ADP-ribose binding, albeit at very high concentrations [67]. In addition, a fragment-based screen showed binding of 2-oxo-1,2-dihydro-4-quinazoline-carboxylic acid (SRI-43750) to the CHIKV macrodomain [66]. 

Importantly, although macrodomains are also present in human proteins, structural and electrostatic differences should permit virus-specific targeting [54,68]. For example, although MacroD2 is the closest human homolog of SARS-CoV-2 with ~30% sequence identity and a similar 3D structure, the amino acids surrounding key residues for catalytic activity are different (Figure 5b), resulting in a less charged pocket [54]. As expected, the P1 pocket is well conserved for ADP-ribose binding, but P2 and P3 pockets are much less conserved and can be exploited for selective drug targeting against the viral macrodomain (Figure 5a). Biochemical data have confirmed that MacroD2 has activities that are significantly different from those of the viral macrodomains. MacroD2 binds much more efficiently to ADP-ribose than coronavirus macrodomains but ADP-ribosylhydrolase activity is reduced [32,54]. Consistent with these structural and biochemical findings, our recently developed ADP-ribosylhydrolase activity assay identified dasatinib as an inhibitor of SARS-CoV-2 Mac1, but not human MacroD2 [54]. Our data demonstrate the feasibility of identifying selective inhibitors based on ADP-ribosylhydrolase activity, paving the way for screening larger libraries to identify improved macrodomain inhibitors.

## 8. High-Throughput Assays for Compound Screening 

**Figure 6:**
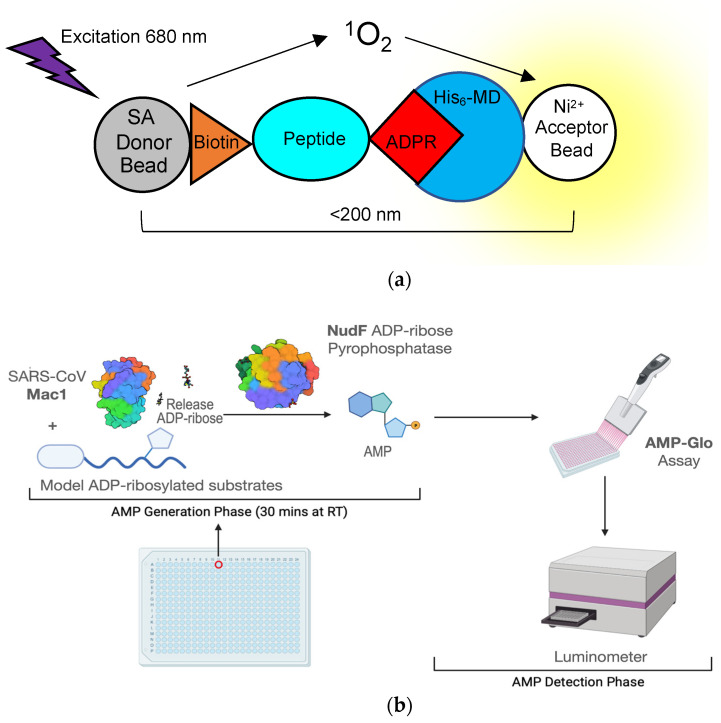
Assays developed to use high-throughput screening for macrodomain inhibitors. (**a**) Cartoon diagram depicting a bead-based AlphaScreen assay for measuring macrodomain interaction with an ADP-ribosylated peptide. SA—streptavidin; Ni^2+^—Nickel; His_6_-MD—Histidine-tagged macrodomain; ADPR—ADP-ribose; (**b**) Schematics of ADPr-Glo assay (see text for more details).

Not only is the macrodomain a suitable target both virologically and biochemically, but high-throughput screening assays are now available to quickly identify hit compounds. These assays can efficiently screen for compounds that can inhibit macrodomain-ADP-ribose binding or macrodomain ADP-ribosylhydrolase activity. One of the first macrodomain high-throughput screening (HTS) assays, developed by Schuller et al., is an ADP-ribose displacement assay using AlphaScreen technology [69]. It was initially described in a screen used to identify inhibitors of the 2nd macrodomain of PARP14 and has since been used for the SARS-CoV-2 macrodomain [67]. AlphaScreen is a bead-based, non-radioactive **A**mplified **L**uminescent **P**roximity **H**omogenous **A**ssay, where a “donor” bead converts ambient oxygen to singlet oxygen, which interacts with an “acceptor” bead generating chemiluminescence at 370 nm and in turn activates additional fluorophores in the bead with emission at 520-620 nm. To give off a signal, the two beads must be in close proximity to each other or the singlet oxygen will go undetected. To make this assay suitable to measure macrodomain-ADP-ribose binding, a peptide was developed that has a biotin molecule attached to one lysine and a non-hydrolysable ADP-ribose on another lysine. Histidine-tagged macrodomains that are bound to this peptide will interact with a donor streptavidin bead and an Ni^2+^ acceptor bead, which will then give off the light signal (Figure 6a). Another ADP-ribose binding assay recently developed by Sowa et al. is a FRET-based assay [70]. In this assay, a YFP-tagged Gα_i_ protein-based peptide is ADP-ribosylated by pertussis toxin at a cysteine residue, which cannot be hydrolyzed by viral macrodomains. This protein is then incubated with a CFP-labeled macrodomain, which will then give off a FRET signal upon binding. Using this assay, the authors identified suramin as a non-specific inhibitor of multiple macrodomain-containing proteins which, interestingly, was previously shown to inhibit alphavirus replication in cell culture [71]. 

In addition, the binding of ADP-ribose or chemical compounds to the macrodomain can be indirectly assessed by differential scanning fluorimetry (DSF). DSF involves the use of a fluorescent dye (e.g., SYPRO Orange) with affinity for hydrophobic portions of proteins, which are exposed as proteins unfold. Binding of ADP-ribose to the macrodomain reduced the increase in fluorescence upon heat denaturation, indicating an increase in the melting temperature, and thus the stability of the SARS-CoV-2 macrodomain when bound to ADP-ribose [32,58]. Virdi et al. used this assay to screen ~2500 compounds for their ability to alter the melting temperature of the macrodomain. This screen identified nucleotides, steroids, antibiotics, and benzimidazoles as potential macrodomain binders. Molecular docking experiments suggested that some of these compounds may interact in the ADP-ribose binding pocket of the SARS-CoV-2 macrodomain [58]. However, many compounds influence the observed protein melting temperature by either quenching fluorescence, increasing fluorescence, or interacting with the reporter dye when mixed with SYPRO Orange, and may therefore be scored as a false positive or false negative in a DSF screen [58].

Measurements of macrodomain ADP-ribosylhydrolase activity have historically relied on gel-based autoradiography and Western blot assays that are not practical for screening large numbers of compounds [26,29]. More recently, our group developed a novel assay that measures ADP-ribosylhydrolase activity in a high-throughput, luminescence-based format called ADPr-Glo (Figure 6b) [54]: First, ADP-ribose is released from a defined MARylated substrate by the macrodomain ADP-ribosylhydrolase of interest. Second, the phosphodiesterase NudF cleaves the released ADP-ribose into phosphoribose and AMP. Finally, AMP is converted to luminescence with the commercially available AMP-Glo kit. This method takes advantage of the substrate selectivity of NudF, which cleaves free ADP-ribose but has no activity toward protein-conjugated ADP-ribose [72]. Therefore, the luminescence signal is controlled by the rate of the ADP-ribosylhydrolase. ADPr-Glo can be performed in 384-well plates with reaction volumes as low as 5 µL, greatly minimizing time and costs compared to traditional gel-based activity assays [28,32]. We established ADPr-Glo conditions for inhibitor screening and multiple macrodomains, including SARS-CoV-2 Mac1, MERS-CoV Mac1, and their closest human homolog, MacroD2. In a pilot screen of the 3233 pharmacologically active compounds, we identified dasatinib and dihydralazine as ADP-ribosylhydrolase inhibitors for both human and viral macrodomains tested. Importantly, dasatinib inhibits SARS-CoV-2 and MERS-CoV Mac1 but not the closest human homolog, MacroD2. The selectivity demonstrates it is possible to discover drugs that specifically inhibit viral macrodomains. Although cytotoxic when used at µM concentration, dasatinib has antiviral activities against SARS-CoV and MERS-CoV through an unknown mechanism [73]. These proof-of-concept data pave the way for screening large compound libraries to identify improved macrodomain inhibitors and explore their potential as antiviral therapies for SARS-CoV-2 and future viral threats [54].

Finally, Russo et al. also developed an immunofluorescence-based assay to assess the activity of SARS-CoV-2 Mac1 activity in cells [74]. Activation of IFN responses, by treating cells with IFN-γ or the double-stranded RNA mimic, poly(I:C), robustly induces ADP-ribosylation in cells, which can be removed by ectopic expression of wild type, but not catalytically inactive mutant, Mac1. Although the initial screen of a limited set of compounds did not yield any hits, this assay may potentially be used in the future for testing the target engagement of potential macrodomain inhibitors in cells.

## 9. Conclusions

Viral macrodomains offer an attractive antiviral target because they are highly conserved and have structurally well-defined (druggable) binding pockets. Because the viral macrodomain is mechanistically distinct from more common antiviral targets (e.g., viral polymerases or proteases), a macrodomain inhibitor would facilitate development of combination therapies for optimal treatment (as successfully developed for HIV). Besides inhibiting viral replication, macrodomain inhibitors may also boost immune responses that contribute to the recovery from disease [75].

Furthermore, coronaviruses and alphaviruses are prominent veterinary pathogens, so macrodomain inhibitors may also be useful for treatment of animals. While the current focus is on targeting macrodomains for antiviral therapy, drug development efforts may identify compounds that can inhibit human macrodomains as well. These inhibitors may have important uses in other human diseases such as cancer, metabolic disorders, and inflammatory diseases. In addition, first-generation macrodomain inhibitors may also serve as tools to probe pathways regulated by ADP-ribosylation that may be attractive novel targets for development of therapeutic interventions.
